# Assessing Pb-Cr Pollution Thresholds for Ecological Risk and Potential Health Risk in Selected Several Kinds of Rice

**DOI:** 10.3390/toxics10110645

**Published:** 2022-10-27

**Authors:** Mengzhuo Cao, Weijing Zhu, Leidong Hong, Weiping Wang, Yanlai Yao, Fengxiang Zhu, Chunlai Hong, Shanying He

**Affiliations:** 1 Institute of Environmental Resources, Soil and Fertilizer, Zhejiang Academy of Agricultural Sciences, Hangzhou 310021, China; 2 Shanghai Huadi Environmental Technology Co., Ltd., Shanghai 201803, China; 3 Huahuan Testing Technology Co., Ltd., Shanghai 201803, China; 4 School of Environmental Science and Engineering, Zhejiang Gongshang University, Hangzhou 310012, China

**Keywords:** soil, rice species, heavy metals, ecological risk, health risk, thresholds

## Abstract

The expected typical gley moist paddy soil was collected in Zhejiang Province, China, and conventional (XS 134 and JH 218) and varieties of hybrid (YY 538 and CY 84) rices were used for a pot experiment. The effects of exogenous heavy metals lead (Pb) and chromium (Cr) on rice growth and the accumulation of heavy metals in the grains were studied. The results show that heavy metal concentrations in soil and rice grains have significant correlations, and Pb and Cr significantly (*p* < 0.05) inhibited the rice growth (plant height and panicle weight). The potential ecological hazard index (RI) of heavy metals in the soil was 4.88–6.76, which belongs to the grade of “slight ecological hazard”, and Pb provides a larger potential ecological hazard than Cr in the studied region. The thresholds for potential health risks and ecological risks for Pb and Cr were lower than the “Control Standards for Soil Pollution Risk of Agricultural Land (Trial)” (GB15618-2018, China). This work provides the basis for soil pollution control for Pb and Cr and the selection of rice cultivars from Pb and Cr accumulated soils.

## 1. Introduction

With rapid economic and industrial development, the heavy metal contamination in China has become increasingly serious. The heavy metal contaminants can enter into cropland soils and accumulate continuously. Therefore, heavy metal pollution is increasing in agricultural fields. More than 10 million tons of food was wasted annually due to excessive heavy metals in crops in China [1,2].

Pb and Cr are important pollutants in soil [3]. The enrichment of Pb and Cr in soils not only seriously affects the yield and quality of agricultural crops, but also endangers human health through the food chain [4]. Pb and Cr in rice, which is a major food crop in China, have been a serious problem over the past decades [5,6,7,8]. Therefore, it is important to examine the enrichment of Pb and Cr in rice from soil [9,10,11,12].

Zou et al. [13] revealed that low concentrations of Pb(II) (≤0.25 mg/L) and Cr(VI) (0.15 mg/L) promoted rice growth, and high concentrations inhibited rice growth (>0.25 mg/L, 0.15 mg/L). Generally, the increase of Pb(II) and Cr(VI) concentrations in soils leads to their enrichment in rice [14]. However, the current studies of Pb and Cr enrichment in rice still concentrates on the role of Pb or Cr alone, without considering the effect of their combination [15,16]. In addition, there are numerous rice cultivars, and different rice cultivars differ in their ability to enrich for Pb and Cr. Therefore, it is necessary to analyze rice varieties in representative crop production areas for their ability to enrich Pb and Cr, and thus to draw safety limits for Pb and Cr in soil when growing these kinds of rice varieties.

In the present study, four rice species (two conventional and two hybrid rice species) were used to investigate their uptake ability of Pb and Cr from soil. Based on potential ecological risk assessment and health risk assessment (national food contaminant limit standard), safety limits for Pb and Cr in soil were calculated. This work provides the basis for soil pollution control for Pb(II) and Cr(VI) and selection of rice cultivars from Pb(II) and Cr(VI) accumulated soils.

## 2. Materials and Methods

### 2.1. Experimental Materials

The soil used in this work was latent moist rice soil collected in Zhejiang Province, China. The experimental soil properties are shown in Table 1. The heavy metal concentrations were all lower than the quality control standards for soil pollution in agricultural areas (Trial) (GB15618-2018, China).

Rice samples were obtained from Zhejiang Province, China. The experimental rice varieties were hybrid rice (Yongyou 538 (YY 538) and Chunyou 84 (CY84)) and conventional rice (Jiahe 218 (JH 218) and Xiushu 134 (XS134)). The pretreatment steps of the rice seeds were as follows: 30% H_2_O_2_ soaking for 30 min, cleaning, and then soaking for 10 h [17].

### 2.2. Soil Treatment

The concentrations of Pb(II) (lead acetate, analytical grade) and Cr(VI) (potassium dichromate, analytical grade) added in this experiment are shown in Table 2. Four rice varieties were planted in different concentrations of heavy metal polluted soils (5 holes in each pot and one plant in each hole). Four pots were used for four parallel sets of experiments to reduce experimental error.

### 2.3. Rice Growth Conditions and Sample Detection Pretreatment

Soil moisture content (70%) was maintained during the experiment by the regular addition of deionized water. Composite fertilizers (N, P, K = 17:17:17) were applied at tillering and grain filling stages of rice growth. All the other operations were the same as the field planting operation.

It takes about 132 days for rice to mature from transplanting to rice grain. The aerial parts of rice were taken and dried after being cleaned with deionized water before sample detection. Rice grains need to be dried at 80 °C until constant weight, and then grinded (60 mesh) for the Pb and Cr concentration detection.

### 2.4. Pb and Cr Concentration in Soil and Rice Samples

To determine Pb and Cr concentrations in soil, here the soil samples were microwave digested byHNO_3_-HF-HClO_4_, which referred to environmental protection standard (HJ 491-2019) of the People’s Republic of China. HNO_3_ (GR grade), HF (GR grade), and HClO_4_ (GR grade) were from Huadong Pharmaceutical Co., Ltd., China.

For the Pb analysis, a 5 g sample (accurate to 0.001 g) in rice was weighed in a conical bottle, then 10 mL mixed acid (HNO_3_ and HClO_4_ at the volume ratio of 9:1) was added. The sample was soaked overnight and added into a small funnel to be digested in the electric furnace. If the liquid was brown or black, mixed acid was added until the white smoke appeared. The sample digestion liquid was filtered into a 25 mL volumetric flask with a drip tube. The conical flask was washed five times with a small amount of deionized water, and the solution was mixed in a volumetric flask. For the Cr analysis, a 0.2 g sample in rice was weighed in a microwave digestion tank, and 5 mL HNO_3_ was add for digestion. After cooling, the digestion tank was taken out and boiled at 160 °C on a hot plate until <0.5 mL of the solution remained. The digest was transferred to a 10 mL volumetric flask and brought to 10 mL with deionized water. The concentrations of Pb and Cr were measured by an atomic absorption spectrophotometer (TAS-990AFG, Beijing Pu, China). The instrument reference conditions for the Pb analysis were lamp current 10 mA, assay wavelength 283.3 nm, slit 0.7 nm, drying temperature 150 °C, ashing temperature 900 °C, and atomization temperature 1400 °C; for the Cr analysis lamp current 6.0 mA, assay wavelength 357.9 nm, slit 0.2 nm, drying temperature 120 °C, ashing 900 °C, and atomisation temperature 2700 °C.

The GBW100377 brown rice flour national standard material was used for the quality control of the determination of Pb in rice grain. The GBW10048 national standard material was used for the quality control of the determination of Cr in rice grain. Each batch of the samples were individually spiked with a certified sample, and the batch was judged to be unqualified if the test results for the certified sample were outside the scope of the certificate. It needs to be re-assayed until the test result for the certified substance is within the scope of the certificate. The concentrations of Pb and Cr stock solutions, purchased from the National Center for standard materials research, China, were 1000 mg/L.

### 2.5. Ecological Risk Assessment of Pb and Cr Safety Limit Values

The regression equation between the concentration of Pb and Cr and the panicle weight of rice was established. When the panicle weight of the rice decreased by 20%, it was considered to deviate from the normal growth range [18]. The potential ecological risk index (RI) was calculated according to the method of Hakanson et al. [19]:

(1) $$E_{i}=T_{i}\times \frac{C^{i}}{C_{n}^{i}}$$

(2) $$RI=\sum_{i}^{n}E_{i}$$

where “n” is the number of elements analyzed; “i” is the i-th element; “C_i_” is the derived ecological risk limit value (mg/kg) of the i-th heavy metal element; “E_i_” is the potential ecological hazard coefficient of single heavy metal pollution; and “$C_{n}^{i}$” is the reference value (mg/kg). The limits of soil environmental quality assessment indicators (Pb 100 mg/kg, Cr 250 mg/kg) from the national “Soil quality control criteria for soil pollution in agricultural land (for trial implementation) (Trial)” (GB15618-2018) were used. The biotoxicity response coefficients (Ti) of Pb and Cr are 5 and 2, respectively [20]. The potential risk of each heavy metal pollutant was estimated by using calculated RI values, and the ecological risk was categorised. Table 3 shows the association between the risk assessment index and the heavy metal pollution classification.

### 2.6. Estimation of Pb and Cr Safety Limits Based on Health Risk Assessment

The association between Pb and Cr concentration in soil and Pb and Cr concentration in rice grains was fit by using a regression equation (Table 3). Analysis of variance was used to confirm the significance of the regression model (ANOVA). Pb (0.2 mg/kg) and Cr (1 mg/kg) national standards of rice grains for food safety (GB 2762-2017) were used to calculate regression equation. Then, the health risk limit values of Pb and Cr was calculated according to the regression equation.

### 2.7. Data Analysis

The mean standard deviation of plant height, panicle weight, and grain heavy metal content of the rice cultivars were calculated (S.D). The connections of heavy metal concentrations in soils, heavy metal accumulation in rice grains, and rice grain growth were investigated by using the SPSS Statistics v 21.0 software. Then, the differences between different groups were analyzed for significance (significant at *p* < 0.05, highly significant at *p* < 0.01).

## 3. Results

### 3.1. Effects of Pb and Cr on Rice Growth

**Figure 1:**
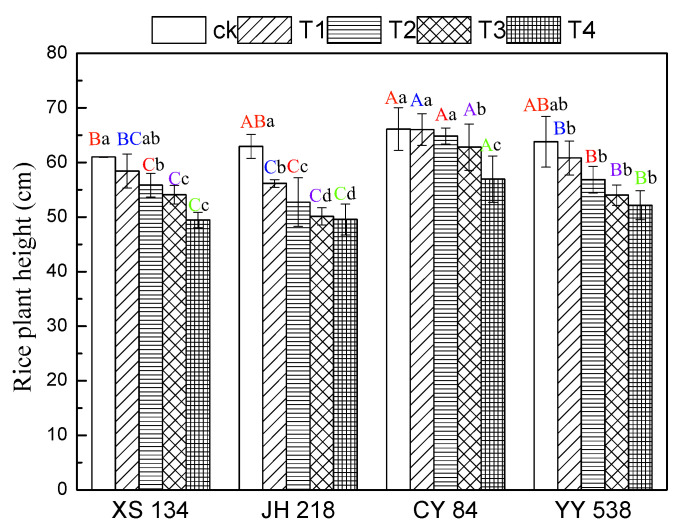
Rice plant height as a function of Pb-Cr contamination. Error bars show the SD of three replicates (n = 3). In the intra-group treatment group, the lowercase letter indicates significant difference between different treatments of the same rice varieties (*p* < 0.05), and in the inter-group treatment groups, the uppercase letter indicates significant difference between different rice varieties under the same treatment (*p* < 0.05).

The height of the rice plant significantly decreased (*p* < 0.05) in all varieties with increasing Pb-Cr concentration (Figure 1). However, the same concentrations of treatments had significant different effects on plant height between conventional rice and hybrid rice (*p* < 0.05). The plant height reduction of the conventional rice cultivars were greater than the hybrid rice cultivars. When the soil was supplemented with Pb-Cr at T4 concentration (Pb = 160 mg/kg, Cr = 500 mg/kg), compared with the control group, the plant height decrease of the conventional rice was 0.7–7.8%, which was higher than that of the hybrid rice. The results showed that the plant height of different rice varieties was significantly different under Pb-Cr stress (*p* < 0.05). The plant height tolerance of the hybrid rice to Pb-Cr was higher than that of the conventional rice.

**Figure 2:**
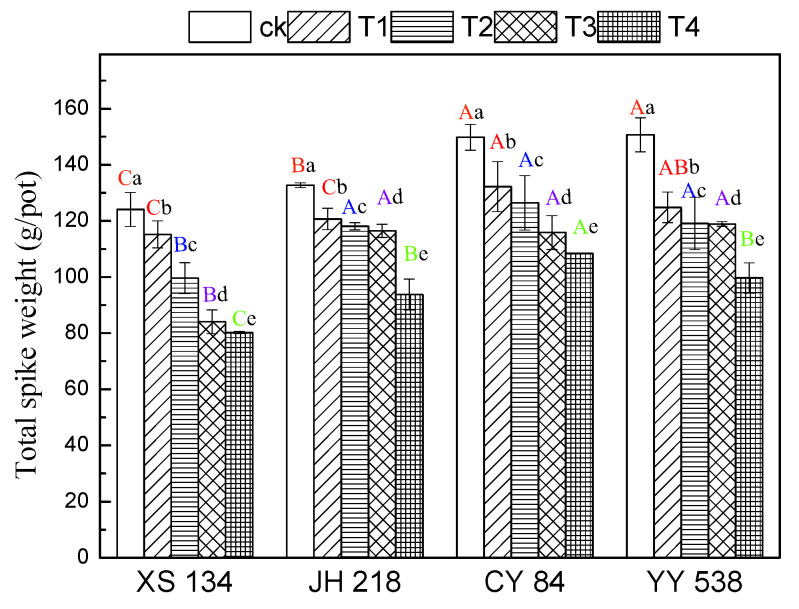
Rice panicle weight as a function of Pb-Cr contamination. Error bars show the SD of three replicates (n = 3). In intra-group treatment group, the lowercase letter indicates significant difference between different treatments at the same rice varieties (*p* < 0.05), and in inter-group treatment groups, the uppercase letter indicates significant difference between different rice varieties under the same treatment (*p* < 0.05).

Rice yield was significantly reduced in all cultivars with increasing concentrations of Pb-Cr concentration (Figure 2). However, under the same treatment concentration, the grain yield of conventional rice and hybrid rice was significantly different (*p* <0.05). Compared with the control (CK), in T4 (Pb = 160 mg/kg, Cr = 500 mg/kg) treatment group, the grain yield of the conventional rice JH 218 and XS 134 decreased by 35.4% and 29.3%, respectively, while that of the hybrid rice CY 84 and YY 538 decreased by 27.6% and 29.05%, respectively. The grain yield of the conventional rice was 0.25–7.8% higher than that of the hybrid rice. The results show that the effect of Pb-Cr on rice yield varies with different rice varieties, and the tested conventional rice was more significantly affected by Pb-Cr soil contamination.

### 3.2. Accumulation of Pb and Cr in Rice

**Figure 3:**
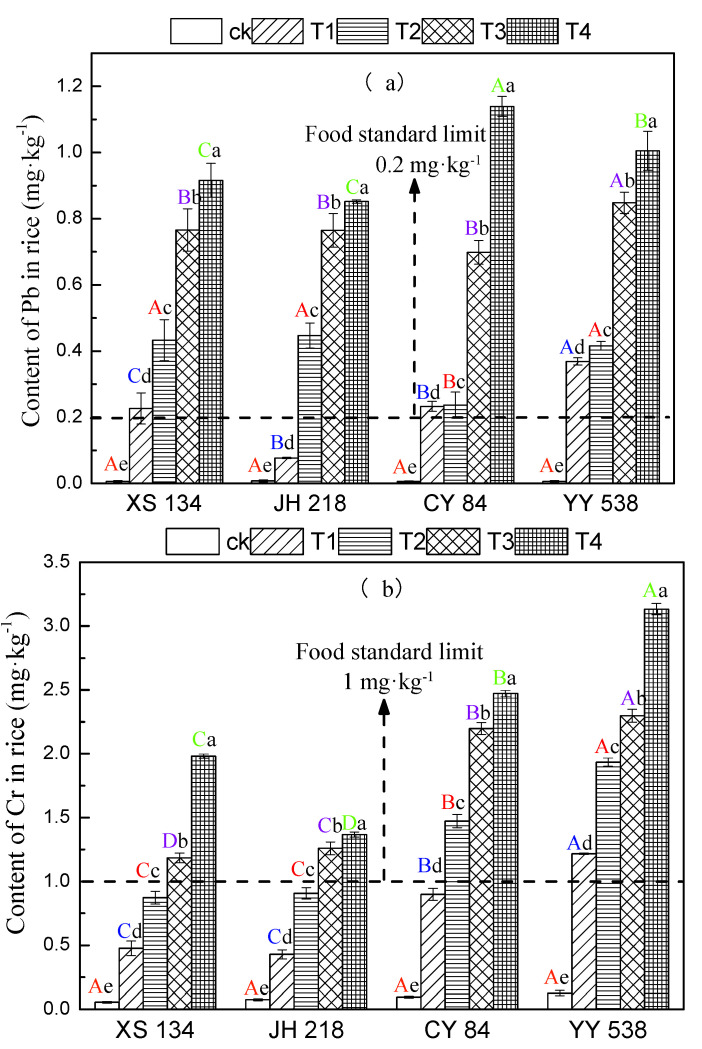
Contents of (**a**) Pb and (**b**) Cr in different rice varieties. Error bars show SD of three replicates (n = 3). In intra-group treatment group, the lowercase letter indicates significant difference between different treatments at the same rice varieties (*p* < 0.05), and in inter-group treatment groups, the uppercase letter indicates a significant difference between different rice varieties under the same treatment (*p* < 0.05).

The concentrations of Pb and Cr in all rice cultivars increased significantly (*p* < 0.05) with increasing soil Pb-Cr concentration (Figure 3). There were significant differences in Pb and Cr contents among different rice cultivars (*p* < 0.05). Under the treatment of T4 (Pb = 160 mg/kg, Cr = 500 mg/kg) concentration, the levels of Pb and Cr in the hybrid rice varieties exceeded the limits of the national food safety standard (GB2762-2017) of 8.86–25.20% and 19.86–56.37%, respectively. The levels of Pb and Cr in conventional rice varieties exceeded 80.11–82.4% and 59.55–68.08% of the limits, respectively.

### 3.3. Ecological Risk of Heavy Metals in Soil

The panicle weight and biomass of all rice varieties are significantly negatively correlated with the contents of Pb and Cr in soil (*p* < 0.05) (Table 4). Among them, the panicle weight of conventional rice reached a significant or extremely significant correlation level with the concentration of Pb and Cr (*p* < 0.05, *p* < 0.01).

When all four rice cultivars showed a 20% reduction in biomass inhibitory, the ecological risk limits for conventional rice grown in Pb-Cr-contaminated soils were all higher than those for the hybrid rice (Table 4). Pb had a higher average RI than Cr based on the single factor potential ecological RI. The ecological risk coefficients (Ei) of Pb and Cr were 1.06–5.00. (i.e., a slight ecological hazard level). The comprehensive potential ecological RI of soil Pb-Cr was 4.88–6.76, indicating a low degree of ecological risk (a minor level of ecological hazard).

### 3.4. Soil Heavy Metal Health Risk Limits

Significant positive correlations (*p* < 0.05) were found between Pb, Cr concentrations in soil and Pb, Cr accumulation in rice (Table 5). However, the uptake capacities of Pb and Cr differed among rice cultivars. Based on the Pb (0.2 mg/kg) and Cr (1 mg/kg) concentration in the National Standard for Food Safety for rice (GB 2762-2017), the regression equation was calculated. The results showed that the concentrations of Pb and Cr in hybrid rice plants were well correlated with the concentrations of Pb and Cr in soil. The following was the soil Pb health risk limits (mg/kg) for safe rice production: XS 134 (59.67), JH 218 (72.68), CY 84 (55.12), and YY 538 (49.06). XS 134 (175.23), JH 218 (193.42), YY 538 (126.23), and CY 84 (94.50) were the Cr health risk limits (mg/kg). The limits of Pb and Cr in conventional rice cultivation were larger than those of the hybrid rice, according to the simulated equation of Pb and Cr limits (Table 5).

## 4. Discussion

### 4.1. Effect of Pb-Cr Complex Pollution on Rice Growth

Rice production and plant height are crucial indicators of rice growth. Rice plant height and panicle weight were considerably hindered by increasing soil Pb-Cr level in the present study. Wang et al. [21] discovered that heavy metal concentrations in soil are inversely linked with plant growth and biomass. Some studies have shown that rice tiller number and plant height decrease with the increasing heavy metal concentration in soil. [22,23], due to the damage of heavy metals to the integrity of root cell membrane and the activity of antioxidant enzymes. Therefore, photosynthesis was hindered, resulting in the reduction of plant metabolic activity, and finally the growth of crops was affected [24,25,26]. Therefore, the growth of rice seedlings could be inhibited. Chang et al. [27] found that when the quantity of heavy metals absorbed by rice increased as the soil pollution load index rises, the chlorophyll content of rice leaves decreases. This might be because heavy metals can cause plants to produce excessive reactive oxygen species (ROS), destroy biological macromolecules (such as plant membrane structures, enzyme systems, and protein), and impede chlorophyll synthesis and plant development [28].

Rice growth is mostly influenced by variety differences [29,30]. Plant height and panicle weight of conventional rice were greatly affected by Pb and Cr in the Pb-Cr-polluted soil of the current study, whereas hybrid rice was less affected. These findings revealed that the tolerance of hybrid rice to Pb-Cr was generally strong, but the selection of rice varieties should be based on the types of heavy metals polluted by soil. Previous studies have shown that the plant height, panicle weight, and seed setting rate of different rice types were all suppressed to varying degrees under heavy metal stress [31]. This might be due to the significant difference in superoxide dismutase activity and chlorophyll content among different rice cultivars under the same environment, resulting in different rice cultivars being different in their resistance to compound heavy metal pollution [32]. However, the mechanism of the effect at the molecular physiological and biochemical level of the plant is still not clear and should be further investigated.

### 4.2. Effects of Pb-Cr Complex Pollution on Absorption and Accumulation by Rice

Pb-Cr in soil and rice Pb and Cr contents were found to have strong and extremely significant positive relationships in the present study (*p* < 0.05; Figure 3; Table 3). This is in line with previous studies’ findings that there are significant differences in the amounts of heavy metals accumulated by different rice cultivars [33,34], and hybrid rice has a stronger ability to absorb heavy metals and transport them to the grain than conventional rice [35,36], which is consistent with Figure 3, showing that conventional rice XS134 and JH218 grains exhibit lower uptake capacities for Pb and Cr. This might be due to the gene control of rice’s capacity to absorb and transport heavy metals, or it could be due to heavy metal biological activity changed by soil microbes, resulting in considerable changes in heavy metal absorption and transport by different rice species and organs. [37,38,39]. As a result, one of the most essential approaches to lower the danger of heavy metal intake in polluted areas is to screen out gene variants with strong resistance to heavy metals through genetic breeding.

### 4.3. Soil Safety Limits of Pb-Cr

The investigation of metal safety limits in soil is crucial in maintaining food and environmental safety. The overall potential ecological RI of heavy metals in the soil was 4.88–6.76 for Pb and Cr concentrations of 76.35–129.92 mg/kg and 132.83–220.41 mg/kg, respectively. The potential ecological risk coefficient of Pb was higher than that of Cr (Table 2). The contribution rate of Pb to the overall ecological risk of heavy metals was 78.19–78.69%, indicating that Pb in soil had a significant ecological risk [40]. The heavy metal toxicity was linked to a single factor ecological risk. For the four rice varieties, the ecological risk thresholds for Pb and Cr in soil dropped in the following order: JH 218 > XS 134 > YY 538 > CY 84. JH 218 (99.92 mg/kg), XS 134 (99.21 mg/kg), YY 538 (98.12 mg/kg), and CY 84 (76.35 mg/kg) were the ecological risk limits for Pb. JH 218 (220.41 mg/kg), XS 134 (174.15 mg/kg), YY 538 (167.86 mg/kg), and CY 84 (132.83 mg/kg) were the ecological risk limits for Cr. Pb and Cr ecological risk limits were lower than the national “Control Standards for Soil Pollution Risk of Agricultural Land (Trial)” (GB15618-2018, China).

The results of this investigation reveal that the safe limits of heavy metals in the soil varied significantly amongst rice cultivars (Table 3). The following were the Pb and Cr health risk limitations in the safe production of several rice varieties: JH 218 > XS 134 > CY 84 > YY 538, and the safety limits (mg/kg) of Pb were 72.68, 59.67, 55.12, and 49.06, respectively. The safety limits of Cr were (mg/kg) 193.4, 175.2, 126.2, and 94.5, respectively. Rice types with greater heavy metal safety standards should be planted to guarantee that the heavy metal content of the rice is within safe limits. Therefore, cultivation of conventional rice JH218 is more suitable for soil contaminated by Pb and Cr. The health risk limit of Pb and Cr were lower than the limits of the soil environmental quality evaluation index in the national “Control Standards for Soil Pollution Risk of Agricultural Land (Trial)” (GB15618-2018) (Pb 100 mg/kg, Cr 250 mg/kg). According to the prediction model of Pb in rice of Fan et al. [41], the limit values of Pb in soil were 230 mg/kg and 110 mg/kg at soil pH 5.94 and 7.50, respectively. In this work, when the pH of paddy soil was 5.32, the Pb limit in paddy soil was 72.68 mg/kg. This implies that, the absorption efficiency of a plant varies dramatically with pH even for the same heavy metal. The determined limit value of Pb in soil in this study was 72.68 mg/kg when the soil pH was 5.32. Furthermore, the crop type, organic matter content, and redox potential of the soil all have an impact on the plant’s ability to absorb heavy metals [42,43,44]. Soil heavy metal limitations should be estimated according to the individual crop species and the soil’s physical and chemical features to successfully implement crop safety production in diverse locations.

## 5. Conclusions

(1) Pb and Cr had a substantially stronger inhibitory impact on the conventional rice development (plant height and panicle weight) than the hybrid rice (*p* < 0.05). Pb and Cr absorption capability of the conventional rice types were lower than that of the hybrid rice cultivars. Pb and Cr concentration in both the hybrid and conventional rice surpassed the food safety standard limit levels when Pb + Cr (160 + 500 mg/kg) concentrations in soil were high.
(2) According to the potential ecological risk assessment, the entire potential ecological risk of Pb and Cr in soil was 4.88–6.76, which was compatible with a minor ecological hazard.
(3) The health risk limit of soil Pb and Cr in the typical paddy soil of Zhejiang Province, China were XS 134 > JH 218 > CY 84 > YY 538. The safety limit of Pb(II) was 72.68, 59.67, 55.12, and 49.06 mg/kg, respectively. The safety limit of Cr(VI) was 193.4, 175.2, 126.2, and 94.5 mg/kg, respectively.

## Figures and Tables

**Table 1:** The soil basic physicochemical properties.

Factor		Value
pH		5.56
Organic matter content		1.79%
Cation exchange capacity		19.2 cmoL·kg^−1^
The soil particle compositions	0.02–2 mm	12.3%
	0.002–0.02 mm	45.3%
	<0.002 mm	42.4%
The heavy metal contents	Pb	24.1 mg/kg
	Cr	94.0 mg/kg
	Cu	21.3 mg/kg
	Zn	64.9 mg/kg
	Cd	0.02 mg/kg

**Table 2:** The concentration of applied Pb(II) and Cr(VI).

Treatment	Concentration	
	Pb(II) (mg/kg)	Cr(VI) (mg/kg)
CK	0	0
T1	40	125
T2	80	250
T3	120	375
T4	160	500

**Table 3:** Potential risk assessment indicators of potentially toxic elements pollutants and their grading relations.

RI	E_i_	Ecological Risk Level
<150	<40	Minor ecological hazard
150 ≤ 300	40 ≤ 80	Medium ecological hazard
300 ≤ 600	80 ≤ 160	Strong ecological hazard
600 ≤ 1200	160 ≤ 320	Very strong ecological hazard
≥1200	≥320	Extremely strong ecological hazard

**Table 4:** Potential ecological risk index (RI) and ecological risk limit value of soil heavy metals.

Heavy Metal	Rice	Correlation Equation	R^2^	Ecological Risk Limit (mg/kg)	E_i_	RI
Pb	XS	y = −2.507x + 349.909	−0.906 *	99.21	4.96	
	JH	y = −2.872x + 434.811	−0.902 *	99.92	5.00	
	CY	y = −1.767x + 292.353	−0.974 **	76.35	3.82	
	YY	y = −2.902x + 447.986	−0.916 *	98.12	4.91	
Cr	XS	y = −3.759x + 548.144	−0.933 *	174.15	1.39	
	JH	y = −4.679x + 717.128	−0.934 *	220.41	1.76	
	CY	y = −2.834x + 479.622	−0.920 *	132.83	1.06	
	YY	y = −4.868x + 754.751	−0.857 *	167.86	1.34	
Pb-Cr	XS					6.35
	JH					6.76
	CY					4.88
	YY					6.25

Note: * significantly correlated at *p* < 0.05; ** significantly correlated at *p* < 0.01.

**Table 5:** Estimated soil heavy metal limit values for the study region.

Heavy Metal	Rice	Simulated Equation	R^2^	Soil Heavy Metal Health Risk Limit Value (mg/kg)
Pb	XS	y = 136.824x + 32.31	0.927 *	59.67
	JH	y = 117.972x + 49.09	0.959 *	72.68
	CY	y = 99.274x + 35.270	0.969 **	55.12
	YY	y = 137.587x + 21.545	0.962 **	49.06
Cr	XS	y = 101.082x + 74.159	0.885 *	175.23
	JH	y = 144.597x + 48.801	0.850 *	193.42
	CY	y = 94.690x + 31.527	0.926 *	126.23
	YY	y = 85.175x + 9.275	0.913 *	94.50

Note: * significantly correlated at *p* < 0.05; ** significantly correlated at *p* < 0.01.

## Data Availability

The data used to support the findings of this study are included within the article. Some or all data or models that sup-port the findings of this study are available from the corresponding author upon reasonable request.
